# Supplementary material for: Splice-Junction-Based Mapping of Alternative Isoforms in the Human Proteome
Source: Cell Rep. Author manuscript; Available in PMC 2020 Jan 15. (PMC6961840; doi:10.1016/j.celrep.2019.11.026)
Supplement: 2 [file NIHMS1546469-supplement-2.zip › DF1/PXD009737/TestisLysC/TestisLysC_16_ADD1_PSCITNCLVTSPPPTTAPHK.pdf]

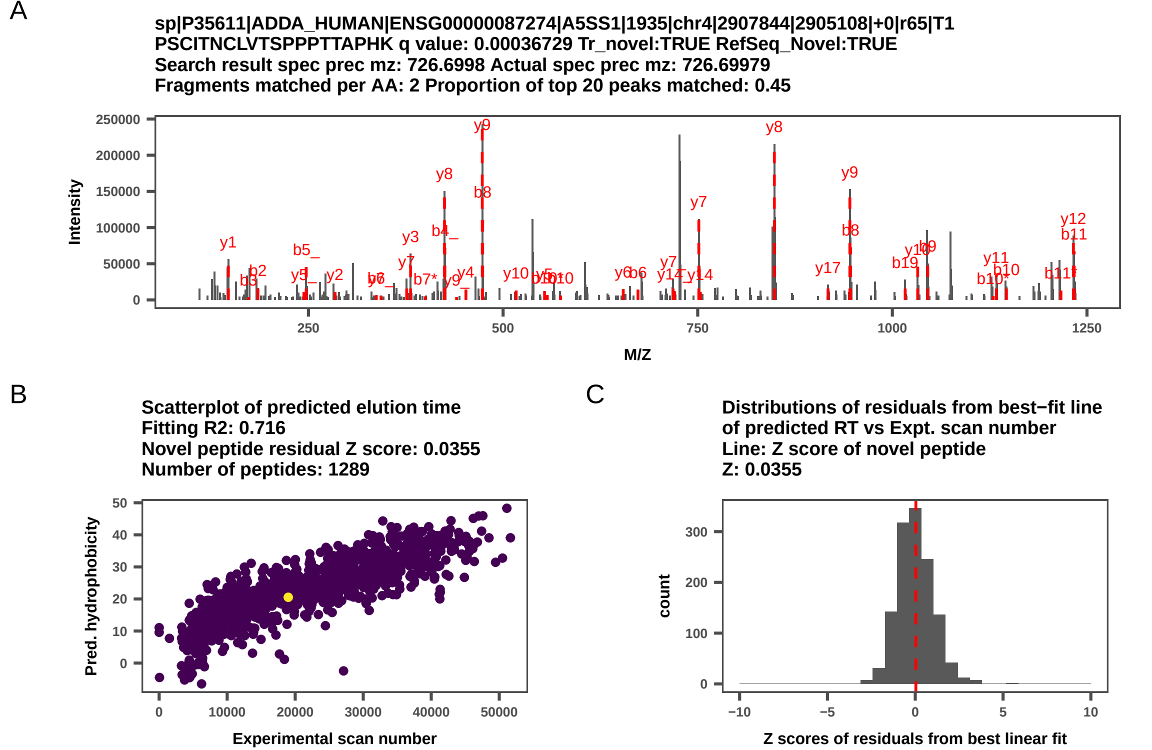
A
sp|P35611|ADDA_HUMAN|ENSG00000087274|A5SS1|1935|chr4|2907844|2905108|+0|r65|T1
PSCITNCLVTSPPPTTAPHK q value: 0.00036729 Tr_novel:TRUE RefSeq_Novel:TRUE
Search result spec prec mz: 726.6998 Actual spec prec mz: 726.69979
Fragments matched per AA: 2 Proportion of top 20 peaks matched: 0.45
Intensity
250000
200000
150000
100000
50000
0
M/Z
250
500
750
1000
1250
y1
b2
b5_
y2
y3
y8
b8
y9
y4
y10
y7
y14
y8
y9
y17
b19
y11
b10
y12
b11
B
Scatterplot of predicted elution time
Fitting R2: 0.716
Novel peptide residual Z score: 0.0355
Number of peptides: 1289
Pred. hydrophobicity
50
40
30
20
10
0
0
10000
20000
30000
40000
50000
Experimental scan number
C
Distributions of residuals from best−fit line
of predicted RT vs Expt. scan number
Line: Z score of novel peptide
Z: 0.0355
count
300
200
100
0
−10
−5
0
5
10
Z scores of residuals from best linear fit
